# The effects of some components on the electrodeposition process used for solar cell applications

**DOI:** 10.1016/j.heliyon.2021.e07554

**Published:** 2021-07-12

**Authors:** Nour Mohammad-Sadik Ali, Ayman Karam, Indrajit Mukhopadhyay

**Affiliations:** a Department of Solar Energy, School of Technology, Pandit Deendayal Energy University; PDEU, Gandhinagar 382007, Gujarat, India; b Institut de Chimie des Milieux et Matériaux de Poitiers, CNRS, Université de Poitiers/ENSIP, 1 rue Marcel Doré, 86073 Poitiers Cedex, France

**Keywords:** Sulfate, Nitrate, Chloride, Electrodeposition, Solar cells

## Abstract

The study has described through the extrapolation method the roles of those precursors' ions as main substances accompanying the progress of electroplating processes that have been used mainly in the deposition of semiconductor thin film and in the fabrication of solar cells. The role of some materials as primary salts have been compared to each other according to their structures, and through the extrapolation method the atomic structures of the metals included in those salts have been reviewed in 3D forms, investigated and compared. The nuances, on the other hand, cannot be denied. However, the study has reached a plausible point of comparison to substantiate the pieces of evidence of these ions’ role in the aqueous solitons. Definitely, the aim is to build up the ultimate steps to finally disclose the essential role of some inorganic or organic compounds in the deposition solution, claiming a step ahead for particular purposes about some elements in the periodic table. Basically, the study cannot rebuke that the available data play an innate part in this study and the next investigating steps in the future. This attempt has somehow illustrated the role of sulfate, nitrate and chloride as accompanying ions in the major salts that have been used to get the desired results in solar cells fabrications. Also, the study has confirmed the basics of mechanisms in which those ions could be compared to each other. For instance, sulfate, nitrate and chloride ions can compare the final results of some metals electrodeposition according to the positions of those metals in the periodic table when fabricating the solar cells. The thickness or the atomic composition of Cu and Zn deposits can be increased at considerably higher voltages starting from IB to IIB columns, whilst for Ga and In deposits, they can be increased starting from the top to the bottom of IIIA column.

## 1 Introduction

Electrodeposition and vacuum deposition that are complementary to each other are in continues growth in various applications. Mostly, they are considered jostling processes in the sense of this competition includes the usage of both deposition technologies advantages (see Table 1).

**Table 1:** Usage advantages of both deposition technologies; vacuum deposition and aqueous deposition.

Vacuum Deposition:	Aqueous Deposition:
Close toleration: approximate desired resistance.	Less in prices.
Variety of adsorbents options.	More thickness.
Variety of plating options.	Complexity of plating shapes.
	Deposits' features of hegemony and adjustment.
	Dominance of remaining stress.

**Figure 1:**
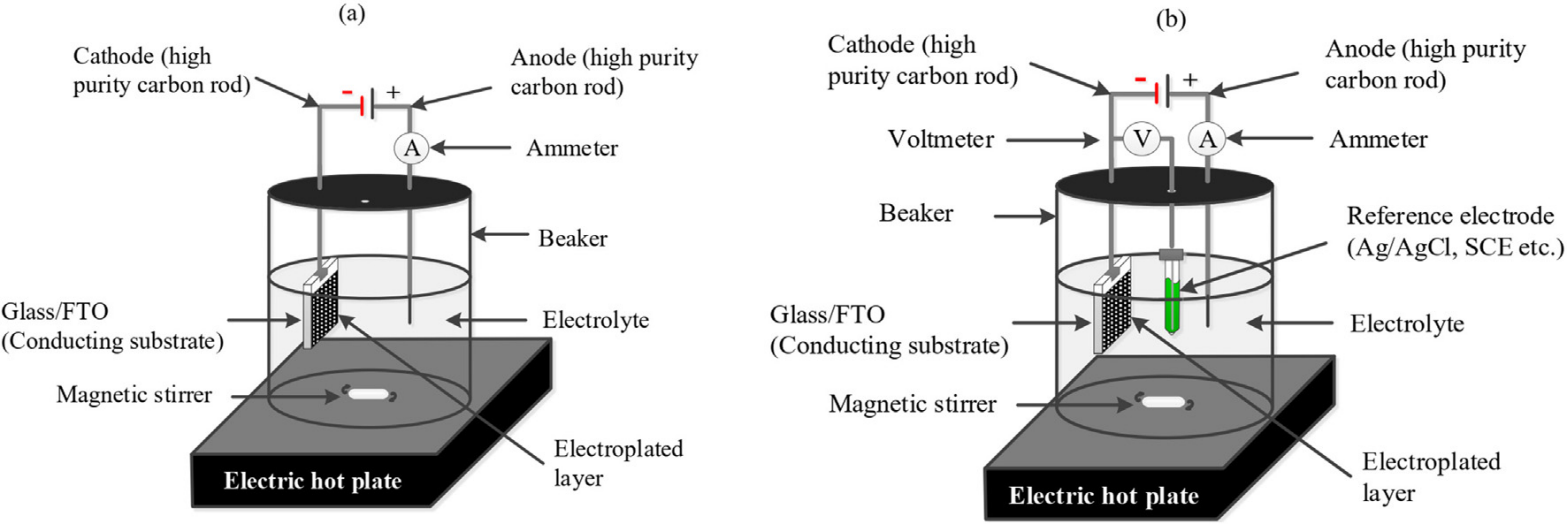
a: Galvanostatic deposition with two electrodes, b: Potentiostatic deposition with three electrodes.

In aqueous deposition, it is essential to either control the current of the bulk solid of the working electrode (galvanostatic mode) or the working electrode potential (potentiostatic mode). Potentiostatic mode is to monitor the bulk current of the solid in order to indirectly determine the interfacial parameters of the solid, and that requires a reference electrode. By the way of contrast, controlling the current is intended in galvanostatic mode. As it is shown in Figure 1a, a standard pattern for galvanostatic deposition process is represented in which two electrodes are used of which comprises a basin, electrolyte, stirrer, hotplate, power supply, working electrode and counter electrode, whereas for a three-electrode setup of the potentiostatic deposition, a reference electrode would be added as it is shown in Figure 1b [1].

**Figure 2:**
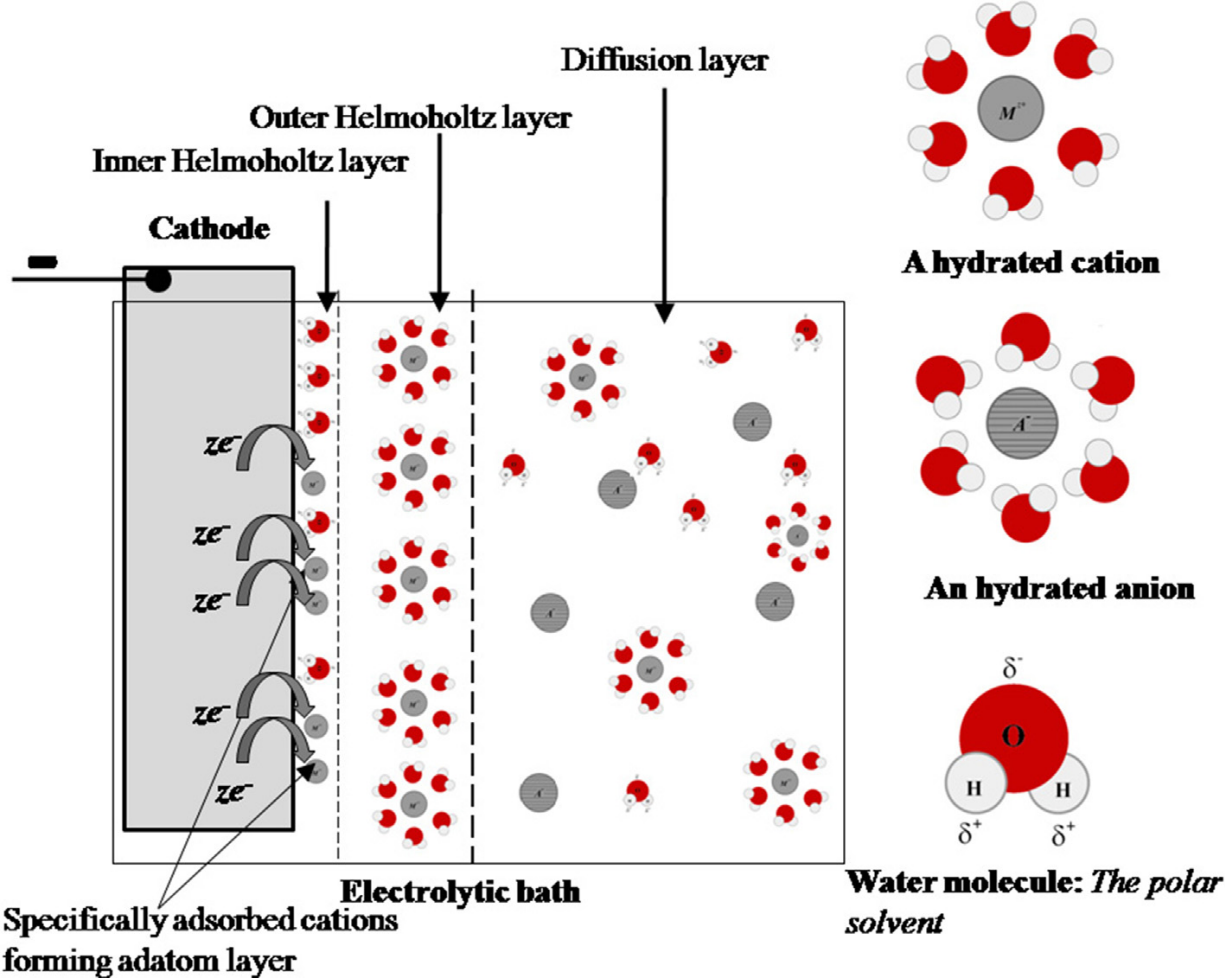
Close section of the electrodeposition process happening on the cathode.

Generally, as long as the electrons are consumed, the cathode processes are classified as reduction reactions, and the oxidations states of the engaging species are reduced. On the other side of the cell, the oxidation reactions happen on the anode in which the electrons are liberated, and the oxidation states are increased. The reactions are autonomous on both electrodes, and each side represents half-cell reactions. However, the number of electrons liberated on the anode and consumed on the cathode must equal each other. Which means the limitation by a condition of material balance takes place. During electrolysis, the complex ions and other kinds would migrate to the anode and are anionic. The mechanisms other than that of simple electron reactions are also involved at the cathode. The specific adsorption effects can occur in the double layer as long as convection and/or diffusion help these complex anions to approach the cathode [2] (see Figure 2).

When short-range interactions between ions and the interphase become significant, ions become specifically adsorbed. It is thought that these ions would infiltrate into the inner layer and may possibly contact the surface. Anions are ordinarily supposed to compose a partial or plenary monolayer [3].

Sundry deposition mechanisms have been suggested in literature, and the major steps consist of the following [4, 5, 6]:

1. Transition of the ions occurs to be deposited on the surface that has a specific nature, which mainly contains edges, incisions, holes and flat surface that introduce essential sites.
2. The adion (partially bound) diffuses and moves across the surface until arriving a growing site where the further process occurs. For instance, dehydration or desorption.
3. Continuous transitions or diffusion proceedings may occur into a texture or get assorted along with other adsorbed ions until they are finally ordained and become a part of a coordinated net.

The locution of adatom or adion confirms the adsorbed state of the atom. Also, since there is a possible fact of that only partial charge transition may occur upon adsorption, an adion remains as a part of its main solvated species, along with a partial charge. Particularly, in the underpotential region, the adsorption processes of metal ions on foreign substrates’ surfaces are distinguished notionally. And this forms the perspective of the diversified techniques used for quantum electronics, catalysis or other applications, where the formation of nanometric items is essential. The islands with well-defined two-dimensional lattice can be built by the adatoms when the coverages are compatibly high depending on the adsorption process on monocrystalline cusps, protrusions and facets with sometimes, in combination with electrolyte anions, the definite lattice can be formed. Adatoms can also form a monolayer. At considerably higher coverages, the reconstruction of an adsorbed monolayer can take place. For example, from a (√3×√3)R30° structure, copper at a Pt(111) surface may be reconstructed in the presence of electrolyte anions as an epitaxial layer Cu(1 × 1) when having a completed coverage [7]. At a given potential, the adions are in equilibrium, both with the solution and with the steps. An equilibrium surface concentration of adions is achieved under these conditions, and Eq. (1) can be written as follows [8]:

(1) $$C_{ad}\left(\text{η}\right)=\;C_{ad\left(\text{η}=0\right)}\;exp\left(zF\text{η}/RT\right)\;$$

Where C_ad (ƞ=0)_ is the adion concentration at the equilibrium potential and different from zero generally. z: electrochemical equivalent. F: Faraday constant (C). ƞ: overpotential (V). R: gas constant (J). T: temperature (˚K).

Adion concentration is not alike across the surface when the incorporation of adions into the grid occurs preferably at the ravels, whereby there is a difference between the electrodeposition mechanism led by the frontal discharge of ions at the growing location and the surface diffusion mechanism. Although it is a one-electron process in the latter case, the electron transfer can be deemed as a two-step process. A partial charge transition takes place upon the species transfer from the solution to the cathode surface, and only because of the final adion combination into the lattice, the charge transition is fulfilled. In literature, arguments in the favour of both hypotheses can be found. The charge transition to a flat surface with a subordinate surface diffusion has been more preferable since it is confirmed by energetic computations. However, the mechanism of direct ion discharge has been supported by plentiful empirical adduced data [8].

## 2 Discussion

1. **Study of the chemical alethiology of sulfate, nitrate and chloride ions:**

Table 2 is the collection of some of those papers that have been done for the sake of the electronic purposes:

**Table 2:** Different aqueous solutions used for electronic purposes using electrodeposition [1].

Material Electroplated	Precursors Used for Electroplating	Outcomes	Ref.
CuInSe_2_	CuSO_4_ for Cu ions, In_2_(SO_4_)_3_ for In ions and H_2_SeO_3_ for Se ions	Ability to grow both p- and n-type material	[9]
CdTe	CdSO_4_ or Cd(NO_3_)_2_ or CdCl_2_ for Cd ions and TeO_2_ for Te ions	Ability to grow both p- and n-type CdTe using Cd-sulphate, nitrate and chloride precursors	[10, 11]
CuInGaSe_2_	CuSO_4_ for Cu ions, In_2_(SO_4_)_3_ for In ions, Ga_2_(SO_4_)_3_ for Ga ions and H_2_SeO_3_ for Se ions	Ability to grow both p- and n-type material	[12]
CdSe	CdCl_2_ for Cd ions and SeO_2_ for Se ions	-	[13]
InSe	InCl_3_ for In ions and SeO_2_ for Se ions	-	[14]
ZnTe	ZnSO_4_ for Zn ions and TeO_2_ for Te ions	Ability to grow both p- and n-type material	[15]
ZnSe	ZnSO_4_ for Zn ions and SeO_2_ for Se ions	Ability to grow both p- and n-type material	[16]
ZnO	Zn(NO_3_)_2_ for Zn ions	-	[17]
ZnS	ZnSO_4_ for Zn and (NH4)_2_S_2_O_3_ for S ions	Ability to grow both p- and n-type material	[18]

Reviewing work [9], CuSO_4_ and In_2_(SO4)_3_ have been compared to each other in order to investigate the accurate role of the sulfate ions in the solution.

**Figure 3:**
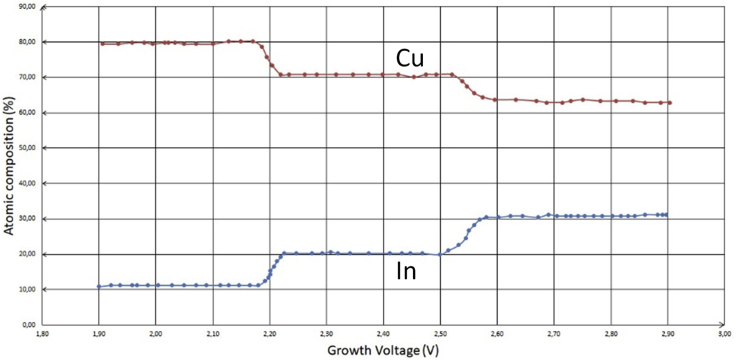
Comparison of Cu and In electrodeposition using XRF technology for thin films at different voltages where a two-electrode system has been utilized [9].

In Figure 3, at low voltages, Cu content is rich, but In content is poor. At higher voltages, In starts to increase in percentage, while the adverse starts for Cu percentage.

Cu (transition metal) > In (metal)

The reason for copper sulfate being in more obstructed movement towards the cathode in the presence of indium sulfate at higher voltages is that indium atom (ion in the solution) has bigger size that allows higher numbers of water molecules through their oxygen atoms to bind with indium atom. As a result of this fact, the bigger atom in size has more charge distribution along its surface. However, that fact does not mean that the atom has more charge positivity. Adversely, it only gives more distribution to that charge on the circumference thereof.

**Figure 4:**
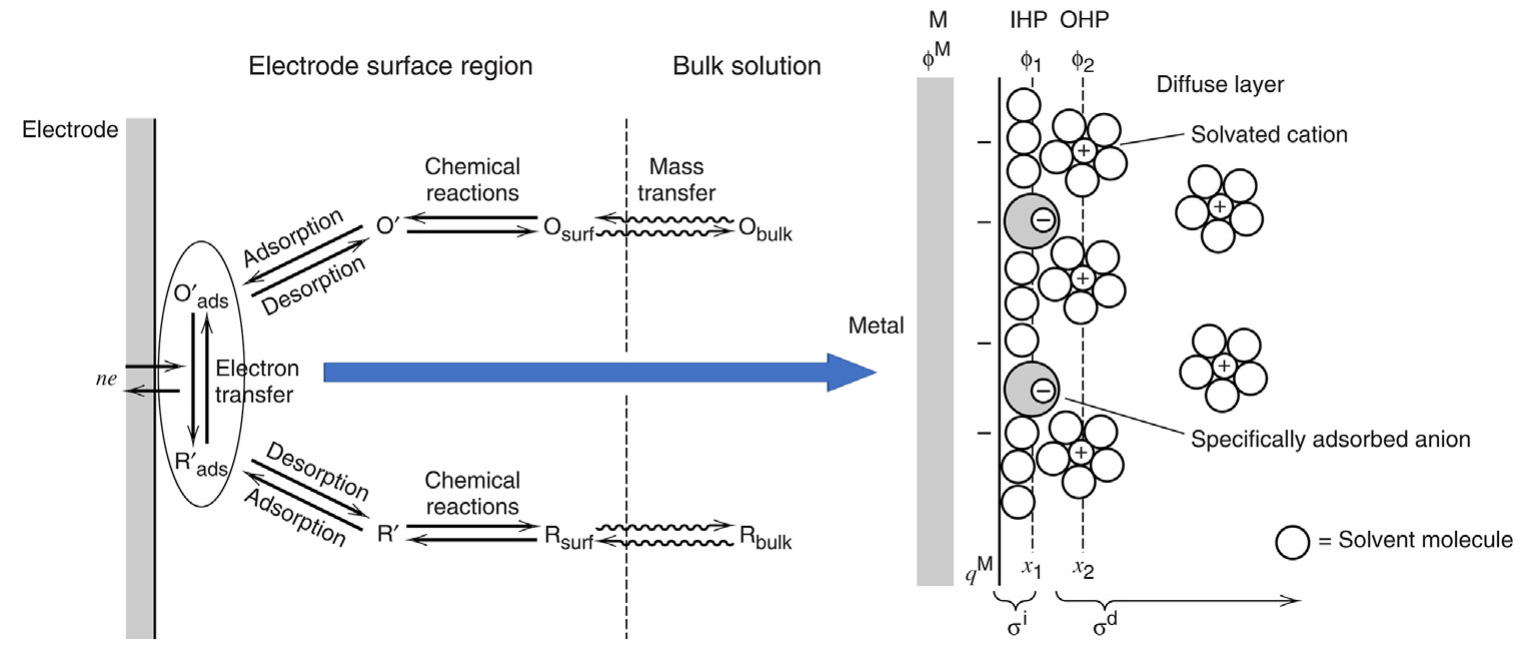
Close section of the electron transition stage near the electrode surface.

In accordance to Heisenberg's uncertainty principle, since the exact position of particles that give the charge property to an atom cannot be known easily, it can be supported that those particles, that are responsible of the charge possession, can attract more water molecules. On the other hand, in order to explain the reason behind more attraction of copper sulfate molecules toward the cathode at low voltages, two facts can be fundamentally significant in this case. First, the low negative charge of the cathode can only attract the smaller hydrated molecules or ions. As it is revealed in Figure 4, the species transfer from the bulk towards the cathode on the left, and the final situation is as represented on the right where the cations are in Outer Helmholtz Plane (OHP) and the solvent particles are in Inner Helmholtz Plane (IHP). Secondly, the interstitial obstruction (3D geometrical structural role). For both facts, other common phenomena are taking place during this process, such as thermal convection and liquid viscosity.

**Figure 5:**
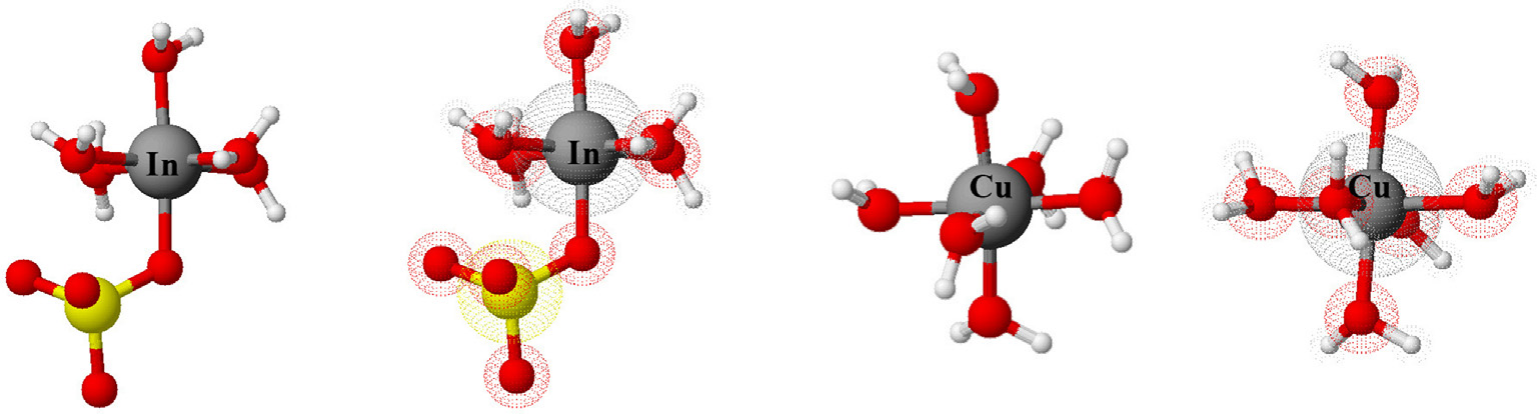
Proximate 3D anthropomorphic molecules for copper and indium salts in the solutions.

It is strongly pertinent to mention that indium ion in water solution forms complexes with water and sulfate. For example, InSO_4_.5H_2_O^+^ and In(SO_4_)_2_.4H_2_O^−^, but copper sulfate exothermically dissolves in water to form aqua complex [Cu(H_2_O)_6_]^2+^. Therefore, indium complexes have more interstitial obstruction than copper complexes because sulfate ions are bigger in size than water molecules (see Figure 5).

**Figure 6:**
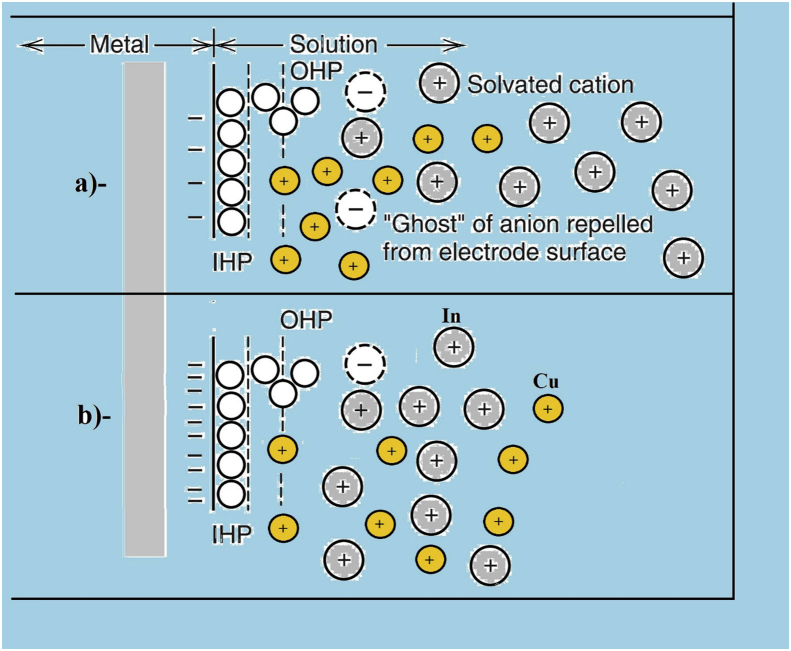
Cu and In ions behaviours in the solution at lower a and at higher b voltages.

Given that, hydrogen atoms with partial positive charge in water molecules are towards the outer phase of the molecules of both hydrated copper sulfate and hydrated indium sulfate. That leads to that indium sulfate, in comparison with copper sulfate, has more attraction to the cathode which has more negative charge at higher voltages (see Figure 6b).

As it can be seen in Table 3, copper has more atomic percentage than indium at lower voltage. However, copper percentage decreases once higher voltages are applied.

**Table 3:** Summary of atomic composition percentages of Cu and In observed using XPS (X-ray photoelectron spectroscopy) for three samples on sputter-cleaned surfaces [9].

Sample	Growth Voltage (V)	Cu (%)	In (%)
a	-2.03	16.8	11.5
b	-2.46	10.5	26.0
C	-2.70	5.8	37.3

**Figure 7:**
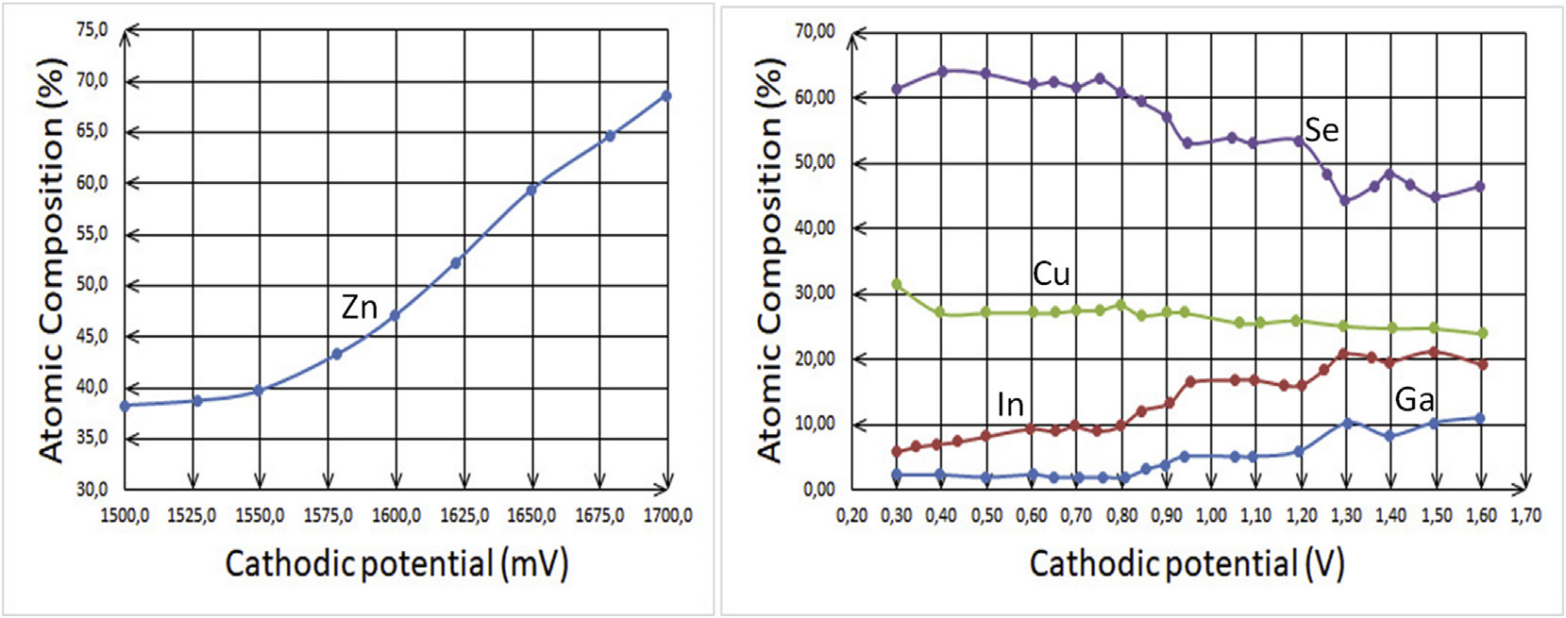
Comparison between two studies to electrodeposit Zn, Cu, Se, In and Ga. a: Atomic compositions of Zn in as-deposited (AD)-ZnTe thin films at different cathodic potentials using 0.015 M ZnSO_4_.7H_2_0 (99.999% purity) as a precursor of Zn [15]. b: Atomic composition of Cu, Se, In and Ga determined by XRF against growth cathodic potentials using a typical aqueous solution of 0.002 M CuSO_4_, 0.004 M In_2_(SO_4_)_3_, 0.004 M Ga_2_(SO_4_)_3_, and 0.004 M H_2_SeO_3_. The material layers were deposited at room temperature and pH = 2 without stirring [12].

Analyzing the situations of other metals, Figure 7 is taken into account. These line-graphs show an interesting phenomenon related to those salts of sulfate in which gallium has a lesser atomic composition percentage than other metals, such as copper, zinc and indium. It can be argued that although indium has a bigger atom in size than other metals represented in this figure and according to that the bigger atom has more reluctance of being attracted towards the cathode, at least at low voltages, it is observed that the atomic composition percentage of indium is higher than gallium at any voltage. Accordingly, the investigation can be first directed towards the compound formulation that gallium represents in the solution. The factual form is that gallium sulfate [Ga(H_2_O)_6_]^+3^ appears in the solution and this formula stays stable as long as the temperature does not increase or when there is no implementation of any of other conditions.

**Figure 8:**
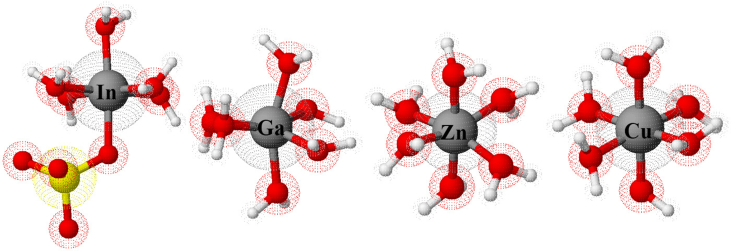
Approximate 3D anthropomorphic molecules of copper, zin, gallium and indium salts in the solutions.

As it is noticeable from the aforementioned formulae with the consideration of central atoms’ sizes included, they are very similar as they do not have different uneven and diversified ligands (see Figure 8). Therefore, this investigation cannot provide the evidence or the justification for what happens in the solutions.

Secondly, the investigation can be towards the analysis and the comparison between the charge distributions on the surfaces of each metal. Hence, although indium atom has bigger size, it does not have the biggest percentage. This means that this investigation again cannot justify the process or provide the reason of having gallium tailing the list of these metals when they are deposited at low voltages; up to 0.8 V.

**Figure 9:**
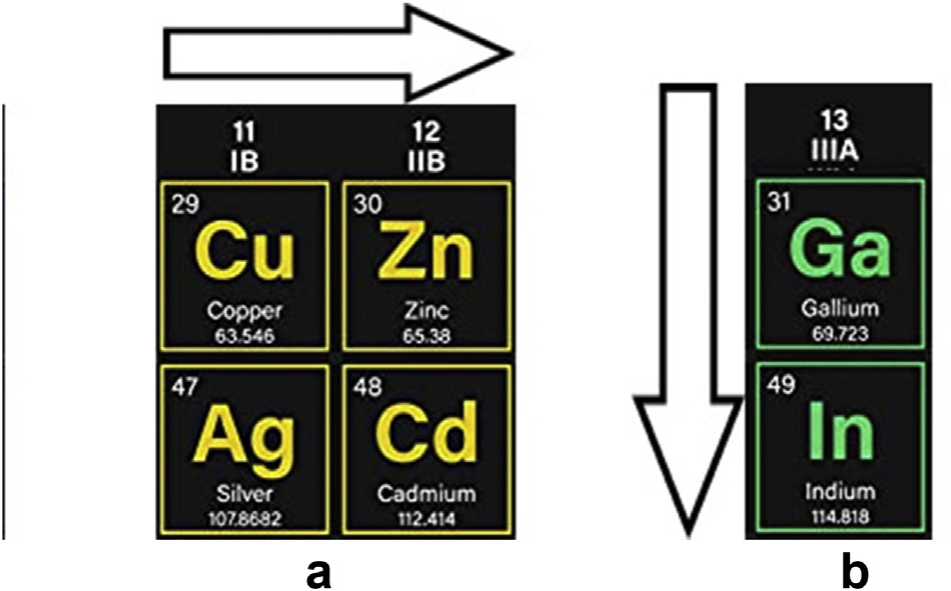
Sulfate salts' role represented as the atomic composition increases with the shift from left to the right side, i.e., a: from IB towards IIB elements. b: The atomic composition increases from top to down in a vertical direction for IIIA elements.

In accordance with the preceding discussion, the outcomes are the following: For transition metals, when using sulfate salts, the atomic composition tends to have bigger values from the left to the right side, e.g. from copper to zinc in the present case. In other words, it goes from IB towards IIB elements in the periodic table (see Figure 9a).

The only observation that can be helpful to elucidate this process is to investigate in more details using the electron configuration of copper and zinc. As the following electron configurations are manifested, the elements that have more electrons in their atomic surface layers reveal more attraction to the cathode, especially at voltages higher than 1.5 V. It seems that 4s^2^ give more charge distribution on the ion surface in the solution than 4s^1^. This leads to more attraction to an active electrode even in the presence of sulfate anions, of course, when other factors and conditions are put aside and not involved. Although 4s has a lesser energy level than 3d, it seems to have a significant role in oxidation states for both ions, copper and zinc, in the solution and it allows zinc ion to have more charge distribution. Therefore, it seems that sulfate ion completely gives the flexibility to the electron configuration to have a dominant role in the solution.

$$\begin{array}{ccc} Atomic\;number & Symbol & Electron\;configuration\\ 29 & \text{Cu} & \left[\text{Ar}\right]4s^{1}3d^{10}\\ 30 & \text{Zn} & \left[\text{Ar}\right]4s^{2}3d^{10}\\ 31 & \text{Ga} & \left[\text{Ar}\right]4s^{2}3d^{10}4p^{1}\\ 49 & \text{ln} & \left[\text{Kr}\right]5s^{2}4d^{10}5p^{1} \end{array}$$

**Figure 10:**
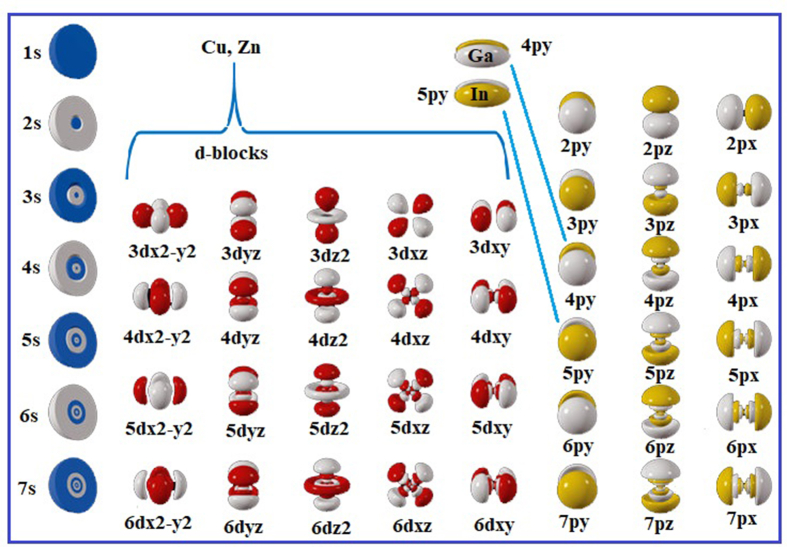
Represented orbitals of the elements in the periodic table; Cu, Zn, Ga and In's orbits have been highlighted and focused on.

Comparing other elements as well, the atomic composition of indium in Figure 7b is bigger in comparison with gallium. This indicates to that the basic metals have more atomic composition when the direction is vertically from top to bottom for IIIA metals (see Figure 9b). It reassures that by moving from 4^th^ energy levels to 5^th^ ones, the charge distribution becomes higher for an ion to be enticed more to the cathode (see Figure 10).

The reason of involving the atomic orbitals in this study is that they reveal that there is an obvious increase in the charge distribution while moving to have additional orbits. This particularly means that the larger surface areas have more vulnerability to be attracted towards the cathode. For instance, it can be possibly argued that 4s orbit when it overlaps 4p orbit of Ga ion, it can be considered as the same as the situation when 5s orbit overlaps 5p orbit of In ion. However, the consideration has to take into account the congregation of all other orbits to finally represent a certain orbital. In other words, in Figure 10, it is so much clear that although the overlapping process of those orbitals could be in the same steps and mechanism, the sizes of the ions are absolutely different due to the fact of having more previous orbits included when moving from one kind of elements to another.

A plausible phenomenon has been noticed. That is the difference that exists on the discussion of the comparison between transition and basic metals in terms of having sulfate-based salts in the solutions for electrodeposition purposes. The main illustration appears to be related to the number of electrons that are responsible of the valence numbers (oxidation states) of an ion, i. e., electrons that exist in surficial orbitals. Cu and Zn ions have one or two electrons in their surficial orbitals, and it is quite suitable to have more charge distribution in comparison with the ions of basic metals, both Ga and In, where the latter ones have three electrons in their surficial orbitals of the 4^th^ and 5^th^ energy levels. This is not compatible enough according to the sizes of the ions in comparison with transition metals. In other words, there are two factors that play an intrinsic role. The first one is the number of the electrons that are responsible of the charge contribution, e.g., if the hybrid orbits (**sp**) are half-full of electrons or less than that. The second factor is diameter of the atom. If it is equal to or less than 2.6 Å, then the attraction towards the cathode will increase when sulfate anions form the basic salts of a metal to be electrodeposited.

Based on the above discussion, it should be noticed that this study does not discuss specific situations in which the potentials of an electrode are chanced in status from an open circuit towards more negative or positive potentials of a closed circuit. The reason is that this study does not parade which element gets reduced first. However, it is intended to evaluate the whole process after the completion of deposition and represents the final results in which the atomic compositions for all elements’ deposits are compared.

**Figure 11:**
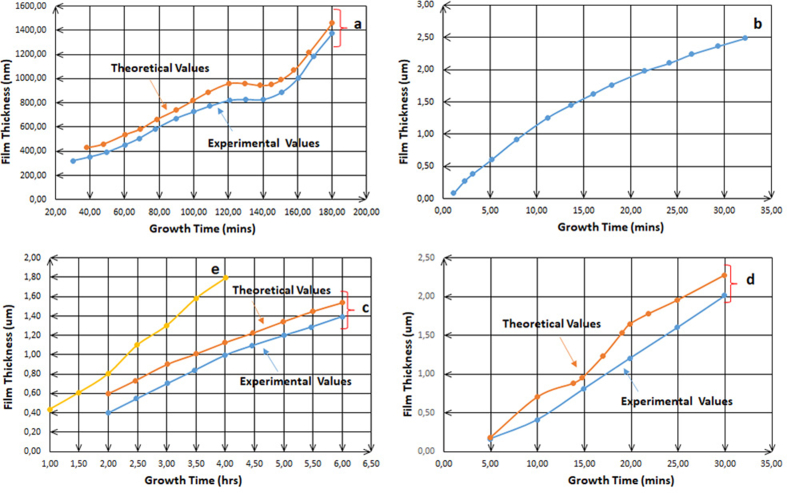
a: Plot of theoretical and experimental thickness estimated for electroplated ZnS films at 1.4–1.5 V [18]. b: Variation of the thickness of ZnO thin films grown at -0.975 V vs Ag/AgCl as a function of deposition time [17]. c: Theoretical and experimental thickness variation with the growth time for the as-deposited CdTe layers grown at 1,253 mV [10]. d: Estimated experimental and theoretical values of thickness for CdSe layers as a function of growth period [13]. e: Plot of the measured thicknesses of In_x_Se_y_ layers versus growth time at 2,123 mV [14].

**Figure 12:**
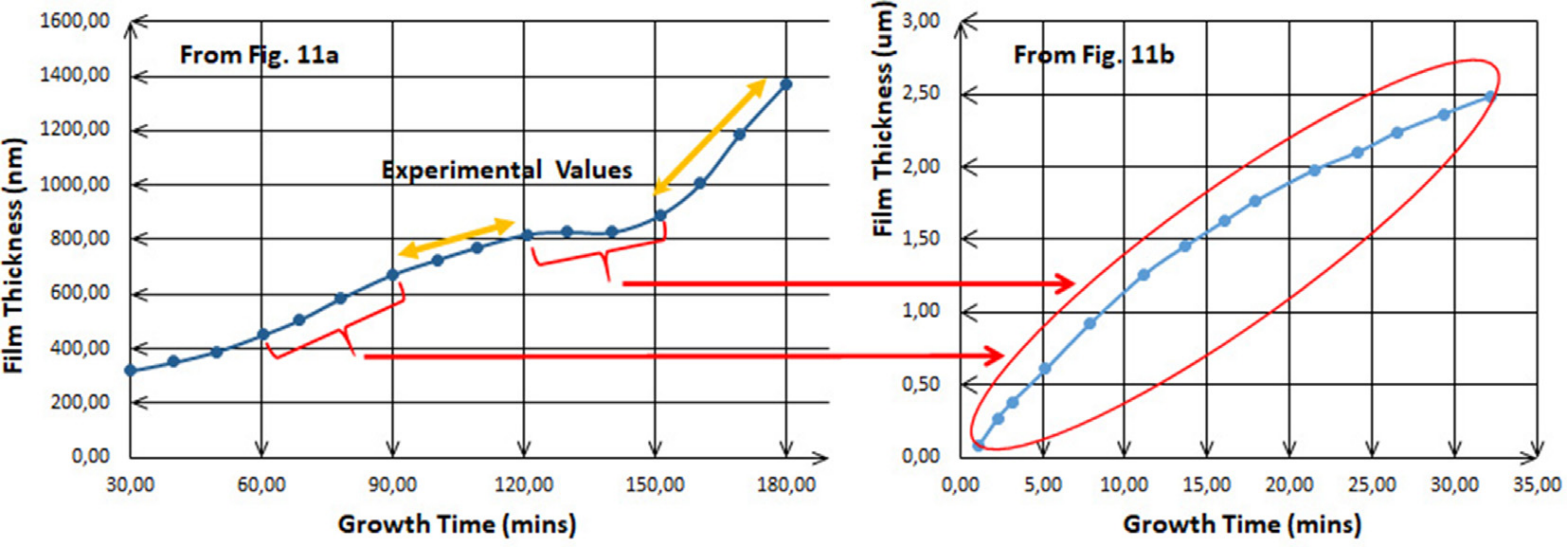
Repetitiveness and periodicity of the curves every 1 h for the electrodeposition of Zn when nitrate and sulfate are used as primary salts in the solution [17, 18].

By analyzing Figure 11, which includes the kinetics of the electrodeposition process of two of transition metals and one of basic metals, these plots are much useful to interrogate what happens in different conditions. Figure 11a shows the electrodeposition of Zn with S at 1.4–1.5 V which is compared with Figure 11b where Zn has been deposited with O at -0.975 V vs Ag/AgCl. It is revealed that the film thickness increases although the conditions are different, and this indicates that the atomic composition also increases with time and is compatible with the results found previously. It has been shown that the thickness of electrodeposited Zn increases with increasing of the voltage value or with time, the primary salts are varied and dissimilar though. In order to compare these two kinds of ions in the solutions prepared for Zn electrodeposition, for the first one; **a**, the salt is of sulfate-based, and for the second one; **b**, the salt is of nitrate-based. Investigating Figures 7, 11a and b, the shapes of those line-graphs are similar, and for the latest two, the curves seems to repeat itself every 1 h as Figure 12 clearly shows the similarity and the repetitiveness.

Figure 12 elaborates the similar mechanisms for both ions, sulfate and nitrate, in the solutions. Also, it undoubtedly remarks that those ions allow the metals ions play the role to being deposited according to their positions in the periodic table, which are in their roles pertinent to the two factors previously mentioned, especially the other conditions are various to each other. This is a strong evidence for that illustration where these results are lastly led to, whether or not the structures of the salts’ ions in the solution have been taken into consideration.

**Figure 13:**
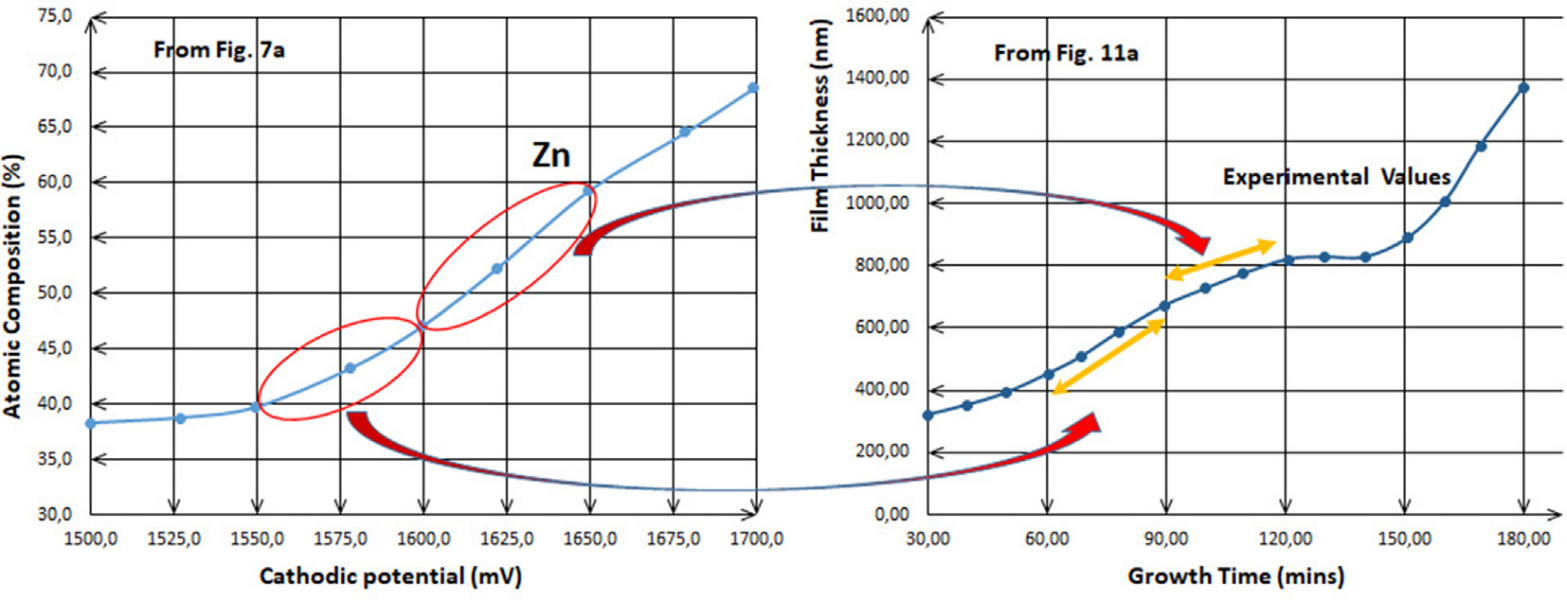
Comparison between Figures 7a and 11a, the similarity and the periodicity of both curves for every 1V as same as for every 1 h.

For further confirmation, the comparison between the two plots in Figures 11a and 7a shown in Figure 13 where both solutions contain the primary sulfate-based salts for Zn electrodeposition has been done. As it is shown, the repetitiveness for both plots is not only for every hourly step, but also for every 1 V step. Thus, this confirms the authenticity of the comparison between curves **a** and **b** in Figure 12. It has to be noted that along with electrodeposition at higher over-voltages, and even with longer duration, the occurrence of secondary reactions on the electrodes takes place. These secondary reactions lead at a certain point to the pairing of electrodeposition with the complex conjugates coming from chain reactions in which few reactions are essential in the solution along with deposition reaction. Upon this fact, it is scientifically quite normal to start having slight differences in the plots at higher voltages or for more time taken.

**Figure 14:**
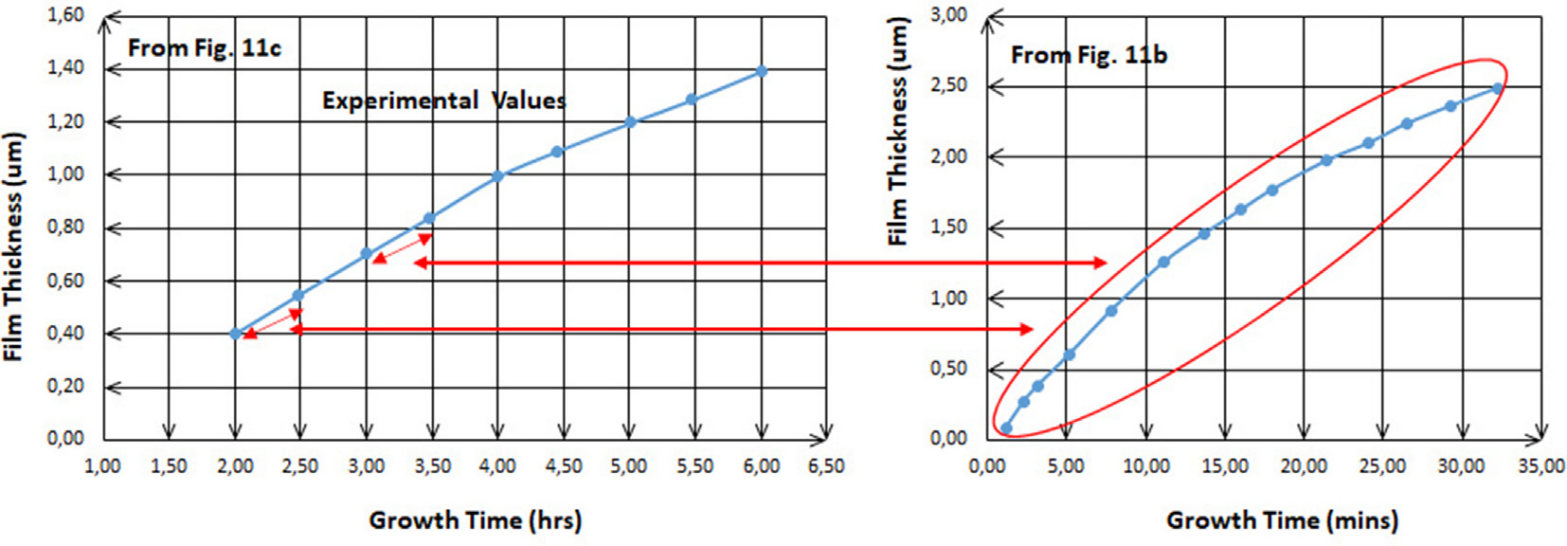
Comparison between Figure 11b and c, similarity and periodicity of both curves for every 1 h.

Similarly, in Figure 14, **c** and **b** plots are of the solutions when cadmium nitrate and zinc nitrate are initial salts and demonstrate the fact of the similarity. This study, of course, is not to accurately and vertically compare between the IIB elements. For example, it is not to compare between cadmium and zinc. That is because the literature studies do not provide enough information or data on solutions used for solar cells. However, as Figure 14 helps to surely reveal the similarity in terms of the interpretation and the exegesis of how both ions, nitrate and sulfate, act in the electrodeposition process. The transitive shape of the relationship between Figures 12 and 14 clearly states that nitrate ions in the solution act in a similar way for cadmium and zin electrodeposition. This demonstrates again that the role of nitrate ions is as the same as that of sulfate ions in the solution.

**Figure 15:**
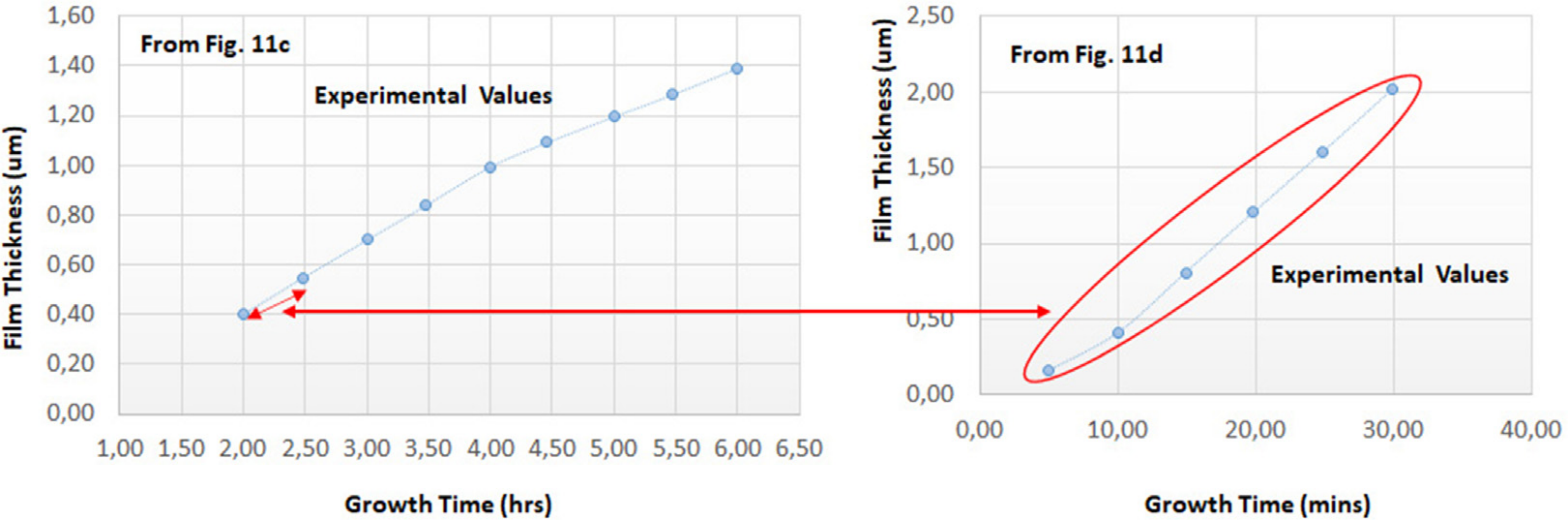
Comparison between Figure 11c and d, similarity and periodicity of both curves for every 1 h.

There are nuances in the experimental values when comparing with other experimental values in different works. However, they are of a considerable extent of similarity. Figure 15 shows that similarity when the comparison is for the same metal; cadmium, with the two different anions; nitrate and chloride, for approximately each 30 min of the electrodeposition process. As long as full 5s orbit is the surficial layer of cadmium atom and full 4s orbit is the surficial layer of zin atom, the role of those orbits has the same analogy. On the other hand, in order to compare cadmium with, for example, indium, the study requires more data. Therefore, in this case, the study cannot have extrapolation steps to confirm the accuracy of the size role. To avoid the confusion, the study confirms that the roles of the three various ions namely sulfate, nitrate and chloride are analogous in their mechanisms to take the atomic structures into consideration.

**Figure 16:**
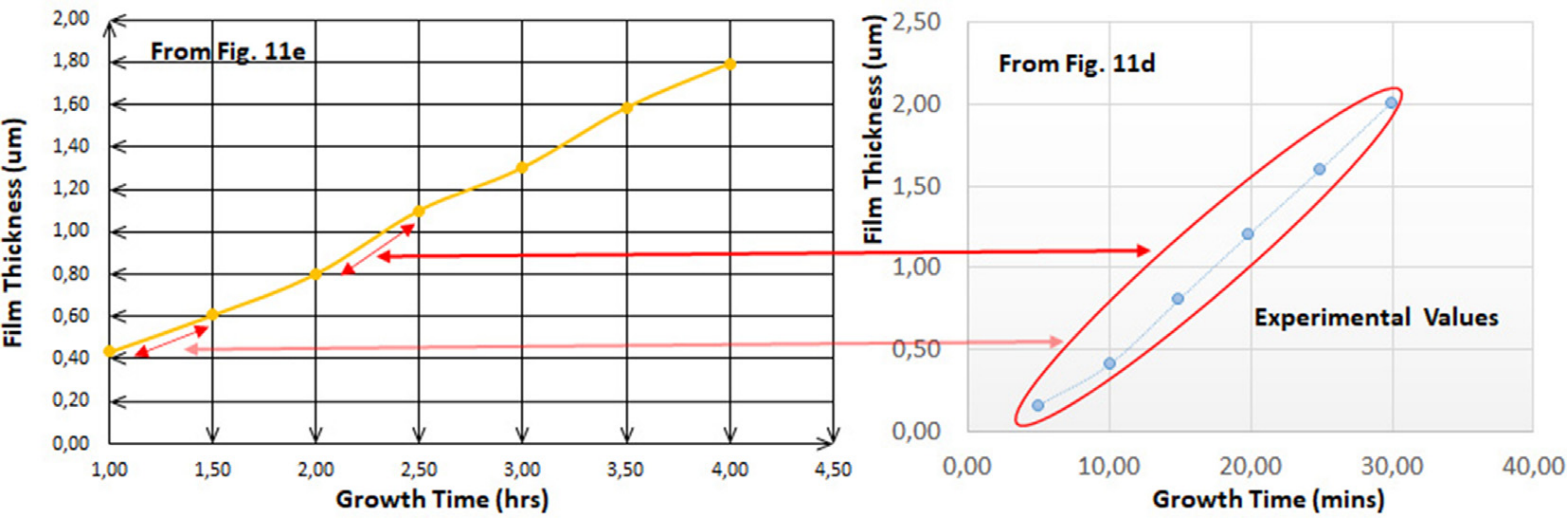
Comparison between Figure 11d and e, similarity and periodicity of both curves for every 1 h.

Another confirmation has been demonstrated through Figure 16. The distinguished property of this comparison is that the deposited thin layers for both plots; **d** and **e**, are the same with the only difference of metal ion, cadmium or indium, where cadmium has been deposited in **d** plot and indium in **e** plot.

2. **Study of organic-compounds effects on sulfate, nitrate and chloride ions behaviours:**

Having the aforementioned elucidations being presented, in further experiments recently, the approval on what has been explained again when the effects of organic compounds are taken into account.

**Figure 17:**
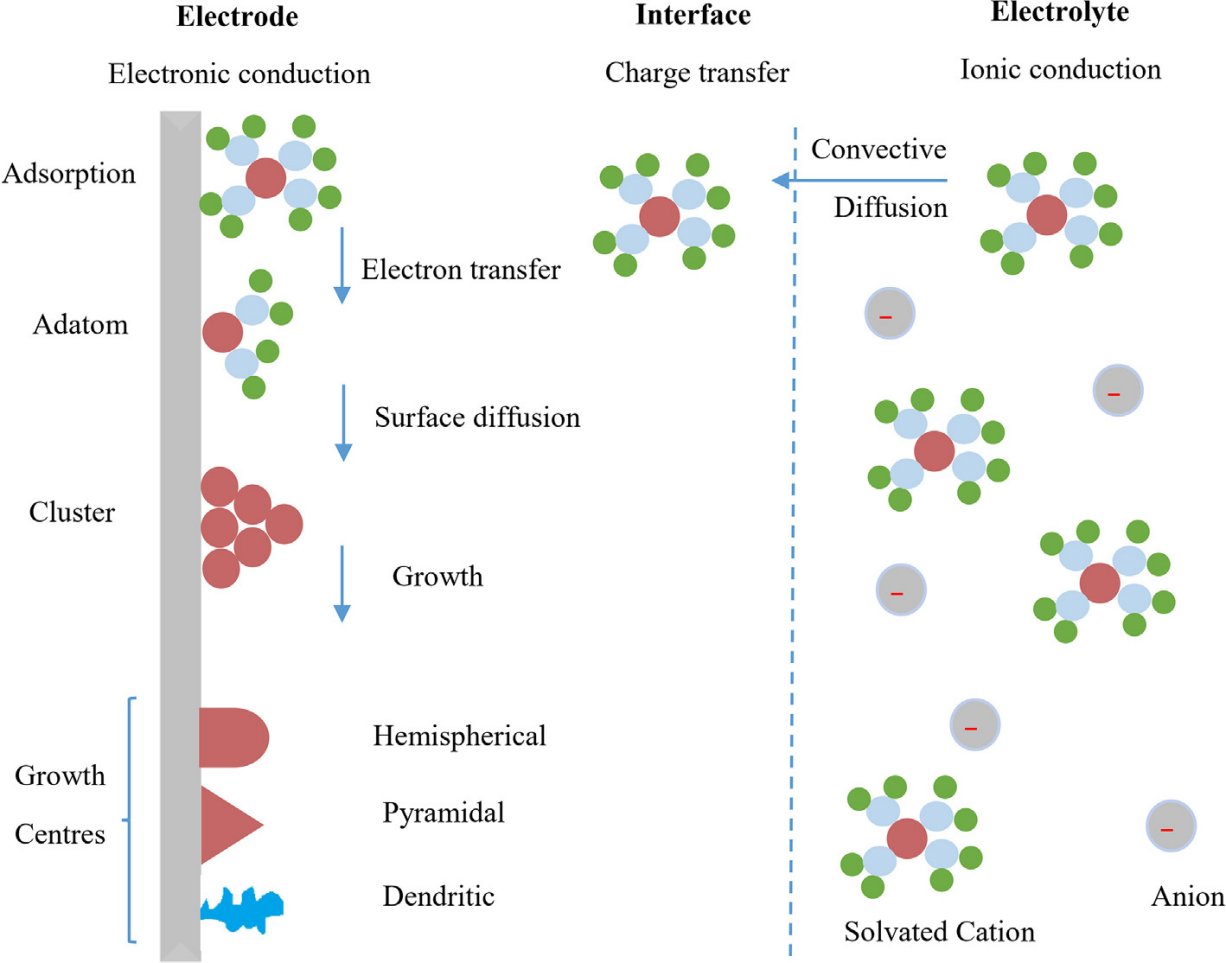
Steps of the metal crystallization during the electrodepositing process from solution bulk until reaching the cathode surface.

Table 4 shows that Cu electrodeposition has been performed by preparing the bath through dissolving CuSO_4_·7H_2_O (99.99%, Alfa Aesar) as the main salt. Also, for depositing In and Ga, InCl_3_ (anhydrous 99.999%, Alfa Aesar) and GaCl_3_ (ultra-dry 99.999%, Alfa Aesar) have been used as main salts in the bath to form another layer of In and Ga on previously deposited layer of Cu. For the complete comparison step, the study must start investigating by looking at the following: In Figure 7b and Table 4, the results are approximately the same when it is found that copper layers have more percentage than both indium and gallium individually, whereas indium has more percentage than gallium. Once again, this bids how both sulfate and chloride act in the solution when they are the main salts. On the other hand, it may be argued that some other ligands have been added to the solution to change the mechanism of the deposition partially or entirely. That is a valid argument in terms of the partial change in the mechanism. However, the partial or the entire change, as the electrodeposition process is not just one step, does not mean to be existed in all the steps of the process. For example, Urea is used to be added to increase the brightness, fineness, homogeneousness and adherence [21]. Figure 17 shows the steps of the electrodeposition process [22].

**Table 4:** Electrodeposition conditions applied to get the metallic thin layers. By using the steps in previous works for getting a wide area of the cathode covered (≈ 4 cm^2^) [19], the following metal ratios have been obtained: [n(Cu)]/[n(In + Ga)] = 0.9 and [n(Ga)]/[n(In + Ga)] = 0.3 [20].

Sample	Cu dep. charge density (C cm^−2^)	In + Ga dep. charge density (C cm^−2^)	Target absorber thickness (μm)
S500	0.33	0.45	≈ 0.5
S1500	0.94	1.28	≈ 1.5

**Figure 18:**
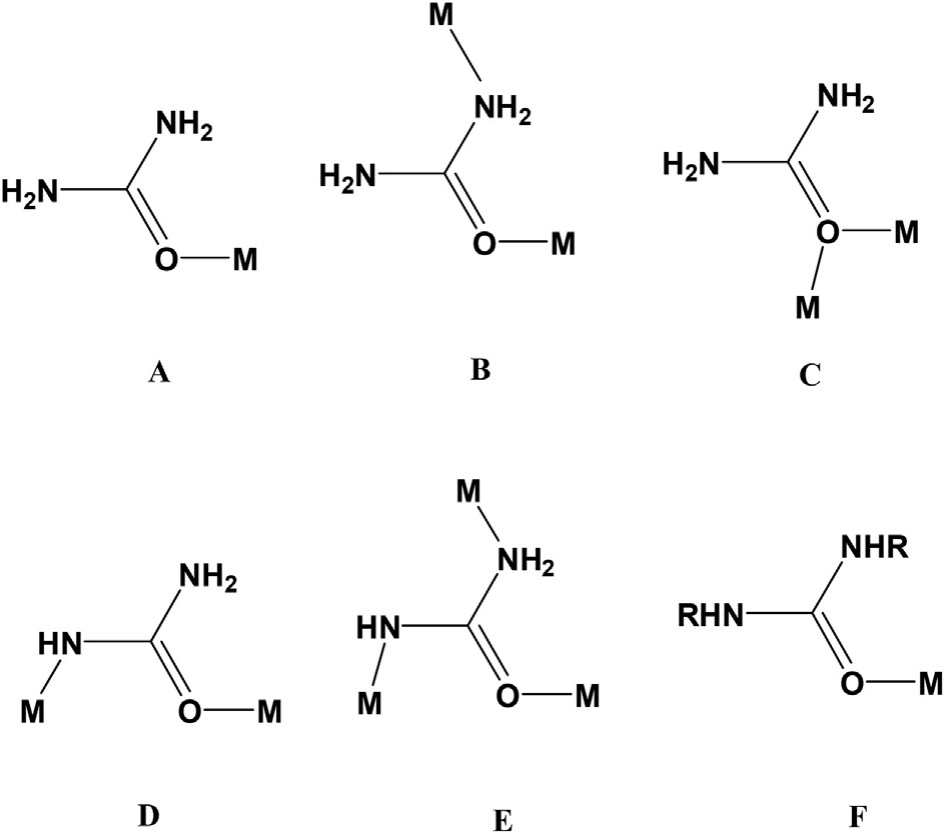
The possibilities for the metals to bond with urea [23].

To clarify the difference of using different ligands in the solution, organic or inorganic, as it is shown in Figure 17, the mechanism depends on the results at the end of the electrodeposition process. To explain in detail, the difference of ligands has to be considered in the following steps: First, the speed and the density of the species in both the convection-diffusion and the charge transfer stages. Second, the whole mechanism of the electron transfer and the subsequent stages, especially beginning with surface diffusion stage. In this regard, the chelation should be taken into account. For instance, the coordination types in Figure 18 and Eqs. (2), (3), (4), (5), (6), (7), (8), and (9) show how these metals chelate with urea where these equations exhibit the expected reactions between some metals including copper with urea at two different temperatures; 25 °C and 60 °C [23].

(2) $${\text{CuCl}}_{2}.2{\text{H}}_{2}\text{O }+2\text{U }+2{\text{H}}_{2}\text{O}\overset{CH_{3}OH,\;25{}^{\circ}C}{\to }\;{\text{CuCl}}_{2}.2\text{U}.4{\text{H}}_{2}\text{O}$$

(3) $${\text{CuCl}}_{2}.2{\text{H}}_{2}\text{O }+2\text{U}\;\overset{H_{2}O/CH_{3}OH\;\left(50/50\%\;v/v\right),\;60{}^{\circ}C}{\to }{\text{CuCl}}_{2}.2\text{U}.2{\text{H}}_{2}\text{O}$$

(4) $$\text{Co}{\left({\text{NO}}_{3}\right)}_{2}.6{\text{H}}_{2}\text{O}+6\text{U}\overset{CH_{3}OH,\;25{}^{\circ}C}{\to }\;{\text{CO}\left({\text{NO}}_{3}\right)}_{2}.6\text{U}+6{\text{H}}_{2}\text{O}$$

(5) $$\text{Co}{\left({\text{NO}}_{3}\right)}_{2}.6{\text{H}}_{2}\text{O}+2\text{U}\overset{{H_{2}O/CH}_{3}OH\;\left(50/50\%v/v\right),\;60{}^{\circ}C}{\to }\text{CO}{\left({\text{NO}}_{3}\right)}_{2}.2\text{U}.4{\text{H}}_{2}\text{O}+2{\text{H}}_{2}\text{O}$$

(6) $${\text{FeCl}}_{3}.6{\text{H}}_{2}\text{O}+3\text{U}\overset{CH_{3}OH,\;25{}^{\circ}C}{\to }\;{\text{FeCl}}_{3}.3\text{U}.3{\text{H}}_{2}\text{O}+3{\text{H}}_{2}\text{O}$$

(7) $${\text{FeCl}}_{3}.6{\text{H}}_{2}\text{O}+3\text{U}\overset{H_{2}O/CH_{3}OH\;\left(50/50\%\;v/v\right),\;60{}^{\circ}C}{\to }{\text{FeCl}}_{3}.3\text{U}.5{\text{H}}_{2}\text{O}+{\text{H}}_{2}\text{O}$$

(8) $${\text{MnCl}}_{2}+3\text{U}+3{\text{H}}_{2}\text{O}\overset{CH_{3}OH,\;25{}^{\circ}C}{\to }\;{\text{MnCl}}_{2}.3\text{U}.3{\text{H}}_{2}\text{O}$$

(9) $${\text{MnCl}}_{2}+6\text{U}\overset{H_{2}O/CH_{3}OH\;\left(50/50\%\;v/v\right),\;60{}^{\circ}C}{\to }{\text{MnCl}}_{2}.6\text{U}$$

It is thus indicated that these metals are still bonded to chloride or nitrate ions even though they are coordinated with urea.

**Figure 19:**
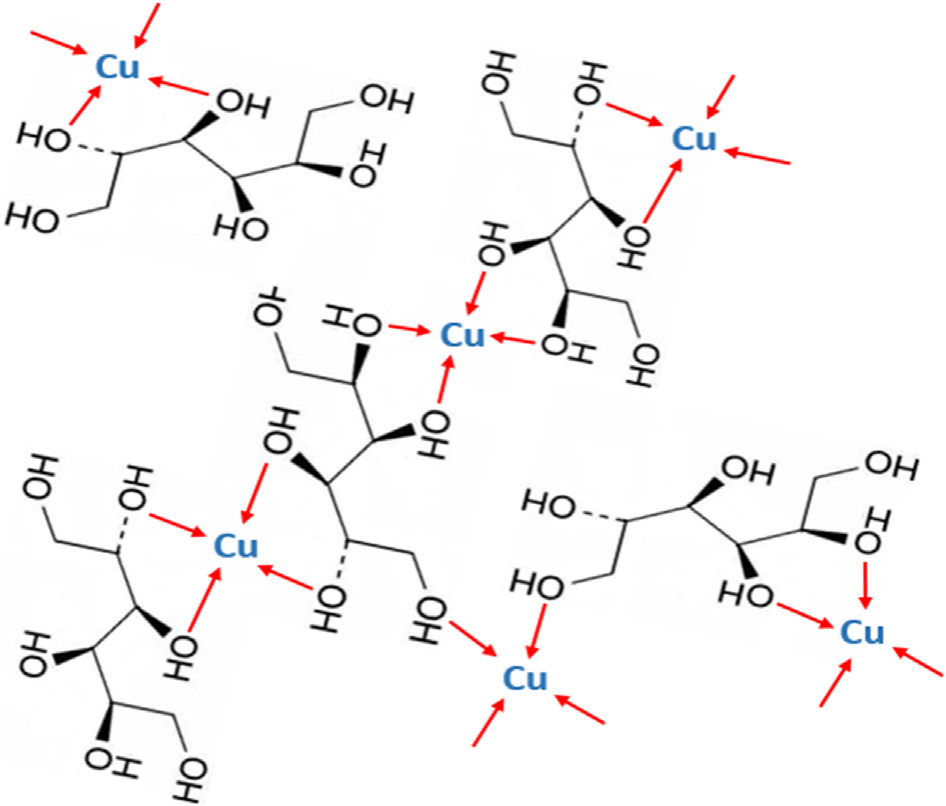
Many molecules of sorbitol are incorporated into copper-hydroxide gel in pH range of 9.5–13. Appearance: gel-like precipitate (dark blue color) [24].

Figure 19 also confirms that those metals are bonded to the ligands by covalent bonds and allows other ions such as sulfate to still be bonded that have been reported recently. The main idea is that the mechanism still works in a general way when such ions, i. e., sulfate, chloride or nitrate are bonded to the metals. By extension through other details related to the effects of other ligands added to the solution, numerous pieces of information are required to be provided. Consequently, long steps of explanations are expected. However, it can be assumed that the nature of coordination with ligands added to the solution besides those metals salts of sulfate, chloride or nitrate has to be considered in the sense of whether those added materials have only secondary or additional effects or have the main effects on a specific mechanism of the electrodeposition process. For instance, some of the ligands are added to chelate with the metals without giving a chance for the main ions included in the salts at the beginning of the process to coordinate with the metals. This might change the whole mechanism of the deposition. Hence, these inorganic ligands work in a similar way for the deposition process of metals. However, the other ligands that are organic compounds in most cases affect the way of organizing the electrodeposition process. The terminology ‘organization’ has many steps included, such as decreasing or increasing the rate of the deposition in different stages, the permissibility of easier or harder electron transfer stage and the overlapping effects of those species during the surface diffusion and growth steps.

At higher voltages, Table 5 reveals the approximate atomic composition of In, Cu and Se. This again confirms the aforementioned comparisons.

**Table 5:** Atomic composition of pulse electrodeposited Cu/In stack and selenized CIS absorbers obtained from EDS analysis [25].

Element (Contents in at. %)	Cu/In stack	CIS absorber
Cu	47.9	22.9
In	52.1	24.6
Se	-	52.5
Cu/In	0.92	0.93

## 3 Conclusion

This work has extrapolated through literature the common ground of behaviours to be laid on as it is the mechanism of some accompanying ions or ligands in the salts that are bonded to the metals in electrodeposition bath, such as sulfate, nitrate and chloride. Further studies for more comparison between these ligands or even between the metals themselves have been aimed. The study has confirmed the basics of a mechanism that those ions can be compared to each other. Thereafter, the effects of those ions on electrodepositing the metals can be established. For instance, sulfate, nitrate and chloride ions can compare the metals electrodeposition processes according to the positions of those metals in the periodic table in order to fabricate solar cells. For Cu and Zn, the increase in the thickness or the atomic composition at higher voltages can be achieved starting from IB to IIB columns, whereas the direction is from the top to the bottom for Ga and In in IIIA column.

## Declarations

### Author contribution statement

All authors listed have significantly contributed to the development and the writing of this article.

### Funding statement

This research did not receive any specific grant from funding agencies in the public, commercial, or not-for-profit sectors.

### Data availability statement

The data that has been used is confidential.

### Declaration of interests statement

The authors declare no conflict of interest.

### Additional information

No additional information is available for this paper.
